# DGLinker: flexible knowledge-graph prediction of disease–gene associations

**DOI:** 10.1093/nar/gkab449

**Published:** 2021-06-14

**Authors:** Jiajing Hu, Rosalba Lepore, Richard J B Dobson, Ammar Al-Chalabi, Daniel M. Bean, Alfredo Iacoangeli

**Affiliations:** Department of Biostatistics and Health Informatics, Institute of Psychiatry, Psychology & Neuroscience, King's College London, SE5 8AF, London, UK; Department of Basic and Clinical Neuroscience, Maurice Wohl Clinical Neuroscience Institute, Institute of Psychiatry, Psychology & Neuroscience, King's College London, London, SE5 9RT, UK; BSC-CNS Barcelona Supercomputing Center, Barcelona, 08034, Spain; Department of Biostatistics and Health Informatics, Institute of Psychiatry, Psychology & Neuroscience, King's College London, SE5 8AF, London, UK; Health Data Research UK London, University College London, London, WC1E 6BT, UK; Institute of Health Informatics, University College London, London, NW1 2DA, UK; Department of Basic and Clinical Neuroscience, Maurice Wohl Clinical Neuroscience Institute, Institute of Psychiatry, Psychology & Neuroscience, King's College London, London, SE5 9RT, UK; King′s College Hospital, Bessemer Road, Denmark Hill, London, SE5 9RS, UK; Department of Biostatistics and Health Informatics, Institute of Psychiatry, Psychology & Neuroscience, King's College London, SE5 8AF, London, UK; Health Data Research UK London, University College London, London, WC1E 6BT, UK; Department of Biostatistics and Health Informatics, Institute of Psychiatry, Psychology & Neuroscience, King's College London, SE5 8AF, London, UK; Department of Basic and Clinical Neuroscience, Maurice Wohl Clinical Neuroscience Institute, Institute of Psychiatry, Psychology & Neuroscience, King's College London, London, SE5 9RT, UK; National Institute for Health Research Biomedical Research Centre and Dementia Unit at South London and Maudsley NHS Foundation Trust and King's College London, London, SE5 8AF, UK

## Abstract

As a result of the advent of high-throughput technologies, there has been rapid progress in our understanding of the genetics underlying biological processes. However, despite such advances, the genetic landscape of human diseases has only marginally been disclosed. Exploiting the present availability of large amounts of biological and phenotypic data, we can use our current understanding of disease genetics to train machine learning models to predict novel genetic factors associated with the disease. To this end, we developed DGLinker, a webserver for the prediction of novel candidate genes for human diseases given a set of known disease genes. DGLinker has a user-friendly interface that allows non-expert users to exploit biomedical information from a wide range of biological and phenotypic databases, and/or to upload their own data, to generate a knowledge-graph and use machine learning to predict new disease-associated genes. The webserver includes tools to explore and interpret the results and generates publication-ready figures. DGLinker is available at https://dglinker.rosalind.kcl.ac.uk. The webserver is free and open to all users without the need for registration.

## Graphical Abstract

**Graphical Abstract:**
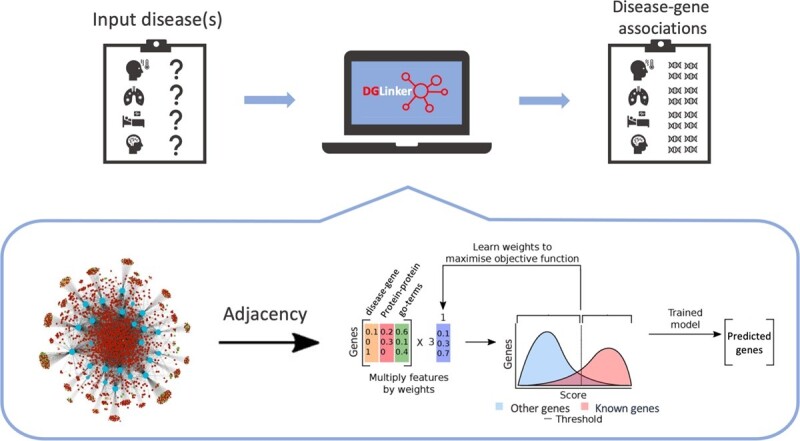
DGLinker: flexible knowledge-graph prediction of disease-gene associations.

## INTRODUCTION

Thanks to the establishment of high-throughput technologies as a common tool in the biomedical field, vast amounts of biological and phenotype information are currently available. Machine learning (ML) is a powerful tool for exploiting this heterogeneous source of knowledge for the prediction of novel associations between biological factors (e.g. genes) and phenotypes. Such predictions can be used for a multitude of purposes including the prioritization of disease genes. Given the large number of targets that high-throughput experiments provide, their individual validation, let alone all the possible interactions between them, is time-consuming and expensive. In some cases, for example for the hundreds of millions of variants from human whole-genome sequencing experiments, this can be prohibitive. In this context, gene prioritization can play an important role (1,2).

The use of ML for the prediction of novel disease-gene associations presents several challenges including the interpretation of the predictions, the selection of appropriate data to generate the model, and an adequate choice of the known disease genes for the training. These are key for usable non-trivial predictions and to avoid model bias (3). Currently available methods generally lack tools for both the interpretation of the predictions and for the evaluation of the model. Moreover, they tend to provide limited flexibility over the data that can be used (1,3–8).

**Figure 1.**
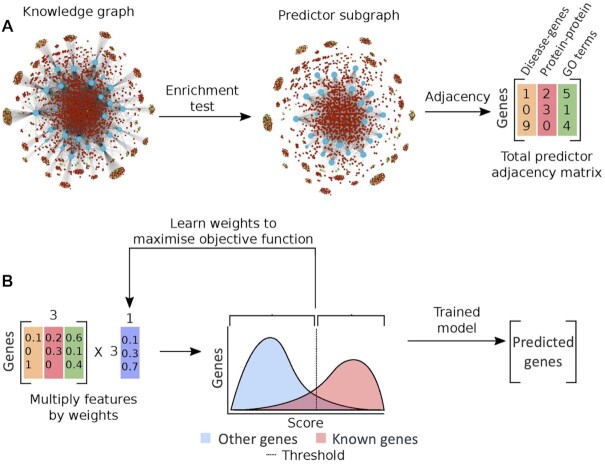
Method overview: The method takes as input a graph of known data related to the prediction task, in this case, gene-disease links, gene functions, and others, and returns a list of predicted edges missing from that graph. (**A**) Starting from a knowledge graph, an enrichment test is used to identify predictive features of the genes known to be associated with the target phenotype(s). The total adjacency of every gene with all predictors of each type (the columns of the matrix) is calculated from the graph. Blue nodes are genes, red nodes are proteins, orange nodes are diseases, green nodes are GO terms. (**B**) The features (adjacency matrix from (a)) are scaled and weighted to produce a final score for every gene. The optimum weighting and score threshold are learned from the set of known associated genes. In other words, to predict new genes linked to a target phenotype, the algorithm compares all genes known to be linked to the target to all other genes and builds a predictive profile based on a weighted combination of existing relationships in the graph. Every gene is then scored for its similarity to this profile. Predictions are made by applying a threshold to this similarity score, with all genes above the threshold predicted as candidate genes. Adapted from Bean *et al.*(9).

We therefore developed DGLinker, a webserver for the prediction of novel candidate genes for human diseases. DGLinker has a user-friendly interface that allows non-expert users to select a customizable set of databases, and use our in-house ML method (3,9) to predict new candidate genes on the basis of genes that are known to be associated with the target disease (method overview in Figure 1).

The webserver includes utilities to explore and interpret the results including a network visualization tool for a graphical exploration of the interactions between the disease genes and other biological factors in the knowledge-graph (KG) that contributed to their classification. It also performs gene enrichment analysis to test the overrepresentation among the predictions of genes associated with specific biological processes (10). Via its user-friendly interface, DGLinker allows users to select a set of databases, including protein-protein interaction, disease-gene (DG) association, transcriptomics, gene function, text mining of scientific literature, and upload their own data for the generation of the KG. The control over the data used in the model can favour the minimization of trivial predictions and hidden biases, factors that can limit the applicability of this class of methods. DGLinker produces a number of publication-ready figures and graphs. The outputs can be downloaded as csv files for use with spreadsheet programs as well as image files of the graphs and figures. On such basis we believe DGLinker to be a novel and promising resource for human disease research in the era of big biological data and precision medicine.

## RESULTS

### Webserver overview

**Figure 2.**
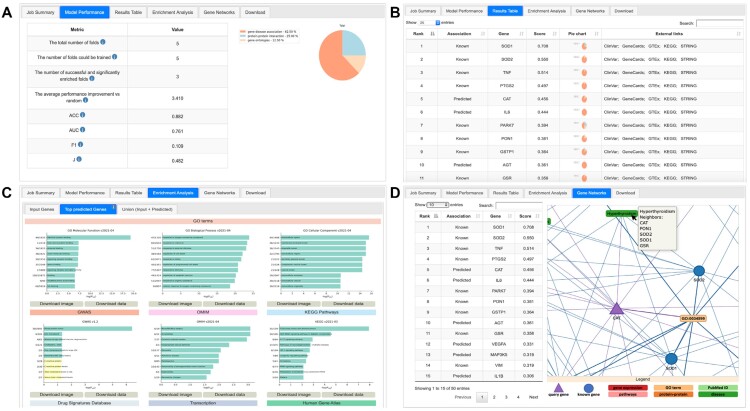
Results visualization and evaluation tabs example. The four panels of this figure display the (**A**) Model Performance tab, (**B**) Results Table, (**C**) Enrichment Analysis and (**D**) Gene Networks for the results of the job example available on the DGLinker website.

DGLinker is a web-based server extension of our previously published knowledge-based ML method (3,9) for the prediction of candidate disease genes. In order to maximise its usability, DGLinker has a user-friendly interface that requires no informatics skills and provides a highly flexible analysis framework that gives the user control over the data used for the generation of the knowledge-graph and the training of the predictive model. Moreover, the webserver provides utilities for the evaluation of the model and the interpretation of the results. These are key aspects that are often overlooked and limit the use of this class of methods in the biomedical field. It is freely accessible and there is no login requirement. The DGLinker pipeline consists of four main steps: (i) specification of known disease associated genes, (ii) selection (and/or upload) of the data to generate the KG, (iii) ML training and DG predictions, (iv) results visualization and evaluation (Figure 2). More details are provided in the corresponding sections below.

### Input options

DGLinker bases its prediction on a set of genes known to be associated with the target phenotype(s). Therefore, the user needs to provide both a list of genes and the phenotypes they are associated with. To do so the following three options are available on the webserver. (i) *Select phenotype(s)*: this option allows the user to provide one or more input phenotypes. DGLinker will automatically retrieve all genes reported to be associated with them in the selected DG databases. Currently, DGLinker includes all disease and phenotype terms, and DG associations from DisGeNet (11), OMIM (12), Clinvar (13) and HPO (14). (ii) *Select phenotype(s) associated genes*: This option allows the user to provide a set of genes and the phenotypes they are associated with. If this option is used, DGLinker will replace the corresponding DG relations in the database with the ones provided by the user. The definition of which genes are associated with a target phenotype strongly affects the predictions and varies greatly among DG databases. This is largely dependent on the evidence used to support their association (3). For example, for genes whose variants can increase disease risk, one might consider the results of a genome-wide association study (GWAS) sufficient while others could require evidence of segregation with the disease in families. Neither choices are right or wrong in general and might depend on the study design and aim. As a consequence, it is very common for a user working in the biomedical field, to have their own curated list of DG associations optimised for their specific study. Input option (ii) is designed to facilitate this common scenario. (iii) *Select genes*: This third and last option allows the user to provide a set of genes without selecting a specific phenotype. This is suitable for studying phenotypes that are not present in the DGLinker database. We recommend the users to search for the target phenotype using option (i) or (ii) before using this option. Where this option is used, it is not a requirement that the associated phenotype is necessarily a disease, for example, a user could specify genes linked to a specific biological pathway or drug response.

### Available databases

DGLinker has a wide range of databases of biological and phenotypic relations available to generate the knowledge-graph (Table 1). These include a selection of 20 databases grouped in following classes: disease-gene associations, protein–protein interactions, gene pathways, expression data, gene function and biological interactions mined from literature. By default, the latest versions of DisGeNet, Gene ontology (15) and IntAct (16) are selected. Users can also upload their own dataset(s). These have to be in comma delimited csv format and include one ‘Gene’ column. The HGNC nomenclature (17) must be used. Currently, there is a limit of 100Mb to upload datasets, however, this limit can be increased, and new databases can be added on demand.

**Table 1.**
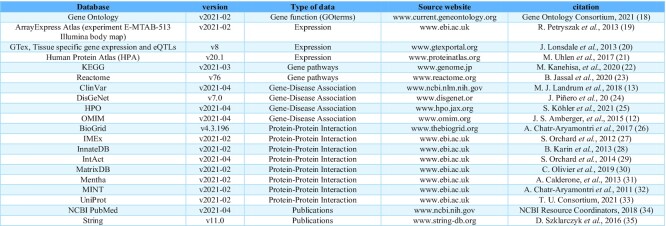
Current set of databases available on DGLinker

### After submission

After submission, the user is directed onto the waiting page where the unique job ID is displayed. This can be used in the homepage to retrieve the job results. If a valid email address was provided at submission, the job ID and a link to the results page are also emailed. The waiting page automatically refreshes every 10 seconds until the job is completed. The user is then redirected to the results page. A standard job with default data sources takes about 10 minutes to be completed. However, jobs can take up to a few hours as the processing time depends on the number of genes and databases used, as well as whether the cross-validation protocol is used. If the cross-validation is selected, DGLinker performs a standard *N*-fold cross validation protocol (36) (where *N* is selected by the user but ≤5) using the input disease genes, and the results are reported in the subsequent model evaluation tab of the results page. Although the *N*-fold cross validation can be a useful tool to evaluate the model performance, it does increase the job processing time by approximately a factor *N*.

### Results page

The results page consists of the following five tabs: Job description, Model Performance, Results Table, Enrichment Analysis and Gene Networks. The Job description tab reports all job details including the job ID, the complete list of the input genes and phenotypes, the number of input and predicted genes and the databases used. In the Model Performance tab (Figure 2A) the user can find a set of metrics useful for the assessment of its quality. These include the results of the cross validation, standard metrics accuracy, F1 and area under the ROC curve (37), and a pie chart representation of the overall contribution of each data source to the model. The Results Table (Figure 2B) displays all the genes, both known and predicted, that the model classified as disease associated, ranked by their score. The score for each gene is its similarity to the learned profile of the known disease-associated genes. The absolute value of the score is not meaningful *per se*, only the relative values between genes of the same model are. Links to external resources, such as Gene cards (38), Clinvar (39), KEGG (40), GTEx (20) and STRING (35) are provided for each gene together with a pie chart representation of the contribution of each data source to the gene score.

The following two tabs, Enrichment Analysis and Gene Networks, are dedicated to tools for the interpretation of the results. The Enrichment Analysis tab (Figure 2c) displays and allows the download of the gene enrichment analysis results that DGLinker generates automatically. This tests the overrepresentation of gene sets from nine databases among the input known disease genes, the predicted genes and their union (10). These databases are the GO gene sets for Biological processes (15), Cellular components and Molecular functions (15), the GWAS catalog (41), the OMIM database (gene–disease associations) (42), KEGG (biological pathways) (40), DSigDB (drug signatures) (43), the Encode and Chea consensus database (transcription) (44,45), and the Human Gene Atlas (46)). The enrichment analysis can help researchers gain insight into the phenotype and biological processes underlying the results. The tab is designed to also allow for a direct comparison between the input known genes and the predictions. In the Gene Networks tab (Figure 2D) the user can visualize the interaction network of each individual gene. The visualization of the individual interaction networks allows for the inspection of the biological and phenotypical factors that contributed to the prediction of a given gene, and of which known disease genes such factors are linked to. Finally, all results, including graphs and figures, gene lists and raw data, can be downloaded as a zip archive from the Download button.

### Comparison to other available DG prediction webservers

By reviewing the available tools for the prediction of novel candidate DG associations given a target phenotype and a set of known associated genes, that (i) have a user-friendly web interface, (ii) are publicly available and (iii) are currently functioning, we have identified two such tools, Phen2Gene (7) and Phenolyzer (4). Additionally, three tools, GeneMANIA (6), MaxLink (47) and ToppGenet (48), despite not allowing the direct input of a target phenotype, can be used to predict DG associations by manually providing a set of disease genes selected autonomously by the user, for example by using an external database of DG associations like OMIM, DisGeNet, or ClinVar (39). In comparison to these tools, DGLinker offers a number of advantages in terms of flexibility and availability of the data to build the model, evaluation of the model and interpretation of the predictions (Table 2).

**Table 2.**
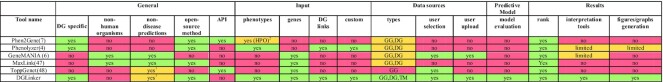
Comparison of available tools for DG predictions. The webservers are compared in terms of characteristics related to their general design, allowed input, data sources used and results section. A traffic light colour system was used for a rapid visual evaluation. ^1^Predictions are not necessarily disease specific, but DG data are used in the model. ^2^Only Human Phenotype Ontology terms (HPOs) are allowed, and they have to be retrieved externally by the user. GG stands for gene–gene (interactions among biological factors), DG stands for disease-gene, and TM stands for text mining (associations mined from scientific literature)

Although GeneMANIA, MaxLink and ToppGenet can make predictions of DG associations, they require the user to perform an extra manual step that is not trivial. Furthermore, GeneMANIA and ToppGenet make use of databases of DG associations for their prediction, as a result, the user-defined input disease genes are likely to largely overlap with DG sets in their databases, leading to trivial and potentially misleading predictions. MaxLink does not use DG databases to build the model, and GeneMANIA is flexible in regard to the databases used so that DG databases can be excluded. However, considering that many human diseases have genetic causes that overlap to some extent or underlie common biological mechanisms, DG databases are a powerful source of information for the prediction of novel DG associations. Therefore, excluding them from the model could impact their performance.

### Performance evaluation

We used the DisGeNet data to simulate prospective prediction performance using a temporal hold-out as an external validation set. We trained the model on DG associations up to and including 2018 and evaluated on all subsequent data as of DisGeNet v7.0 (Table 3). The KG contained DG associations from DisGeNet (v7 2018), protein-protein interactions from IntAct (2020-11-06) and gene function from Gene Ontology (2020-11-17). These three databases are the default setting in DGLinker. Assessing the performance of DG predictions presents several challenges. Due to our limited knowledge of the genetic landscape of most human diseases, complete sets of true positives and true negatives are generally not available. As a consequence, classic metrics such as precision and recall, might not be adequate in this context. Instead, we assessed the model performance using a hypergeometric test for enrichment of newly associated genes in the set of predictions from the model vs the background of all genes in the knowledge graph. Enrichment was considered significant if *P* < 0.05 for hypergeometric test for enrichment following 5% false discovery rate correction. 1131 diseases had at least one new associated gene by 2020 in DisGeNet. For 91% (1024) of these, at least one of the new associated genes was in the KG. For 804 diseases, the model could be trained and made predictions of new disease associated genes. The predictions were significantly enriched for new genes for 184 diseases (22.9%). During training, the model is optimising the J statistic defined as sensitivity + specificity – 1. The top-scoring models (training achieved *J* ≥ 0.9, Supplementary Figure S1) were significant in 42.2% of cases (146/346).

**Table 3.**
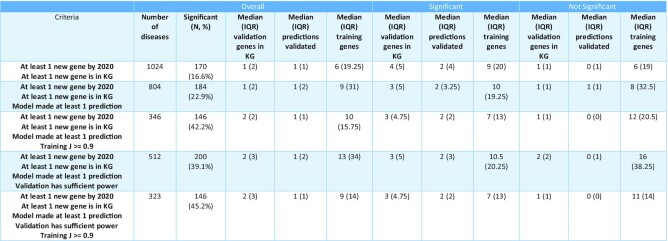
Temporal external validation of predictive performance using DisGeNet. All disease-gene associations in DisGeNet up to 2018 were used to predict associations added by 2020. Significance is determined at threshold *P* < 0.05 after 5% false discovery rate correction for multiple comparisons. ‘Overall’ = values for all models, ‘Significant’ = values for all models that were significant in validation, ‘Not significant’ = values for all models that were not significant in validation. ‘IQR’ = Interquartile range

Given the low number of new associated genes for many phenotypes (median = 2), in some cases even predicting all of them could not result in a significant test. This might result in the underestimation of the model performance. We therefore also reported the overall performance considering only those models that could be significantly enriched given the number of predicted and predictable genes (Table 3, ‘validation has sufficient power’). In these cases, the predictions were enriched for new disease genes in 39.1% (200/512) of all models and 45.2% (146/323) of the models whose training achieved *J* ≥ 0.9. For an overview of how the performance varies with *J* please see Supplementary Figure S1 and Table S1.

We also performed a cross validation study on these diseases. For diseases with at least five known genes in the 2018 data we performed 5-fold cross validation. 599 diseases met this condition, with median 17 known genes (interquartile range = 38.5). For 537 (∼90%) of diseases the cross-validation model was significantly enriched for the held-out genes in at least 3 folds. Although informative, it is important to remark that cross validation is likely to over-estimate the external performance as highly similar genes can be separated across validation folds.

### Software documentation and data availability

The DGLinker website (http://DGLinker.rosalind.kcl.ac.uk) provides an extensive tutorial section in which step by step instructions with figures guide the user through the steps necessary to perform DG predictions and utilize the tools for model evaluation and results interpretation. The Downloads sub-section of the tutorial provides links to all external resources and software used, as well as the links to the GitHub repositories (49) where the KG-ML method code is available under the GPLv3 licence. The ML method is well documented and also available as an open-source python package (https://pypi.org/project/edgeprediction/). The data used and generated in the evaluation of the tool performance are publicly available on GitHub (https://github.com/KHP-Informatics/DGLinker-validation).

### Usage example: Amyotrophic Lateral Sclerosis

The example section presents the results of an application derived from our recent publication (3) in which our method was used for the prediction of novel candidate genes in Amyotrophic Lateral Sclerosis (ALS) using data from early 2019. ALS is a rare (lifetime risk ∼1 in 400 in Europeans), late-onset, fatal disease whose genetic causes are highly heterogenic among patients and largely unknown. Moreover, there is not a complete consensus among ALS experts regarding which genes are implicated with the disease, and as a result, the ALS genes reported in public DG databases vary greatly, ranging from 20 to over 130 (50,51). In this landscape, we have used the DGLinker method to predict candidate ALS genes using four gene sets from as many sources, DisGeNet (101 genes), ALSoD (126 genes) (52), ClinVar (44 genes), a manually-curated list (40 genes) (51) and the union of all these sets (199 genes). In total, 651 genes were predicted. The enrichment analysis highlighted that the predictions were enriched for genes associated with biological processes known to be affected by the ALS pathogenesis, such as angiogenesis (53), lipid metabolism (54), mitochondria activity (55), protein kinase activity (56), superoxide metabolism (57,58), vesicle-trafficking (59), neurotransmitter regulation (60), and with other neurodegenerative diseases for which evidence of phenotypic and genetic overlap with ALS exist, such as Charcot-Marie-Tooth disease, Parkinson′s disease, Frontotemporal dementia, Schizophrenia and Alzheimer's Disease. Moreover, the predicted genes were significantly enriched (*P* = 0.012) for genes that were identified to be associated with ALS in subsequent genetic studies, i.e. they were not yet present in the DG databases used in the experiment. These were *ATXN1* (61), *ATXN3* (62), *SCFD1* (62), *CAV1* (63) and *SPTLC1* (64). Only *ACSL5* (62) and *GLT8D1 (**65**)* were not present among the predicted genes. An extensive discussion and in depth analysis of the predictions can be found in our recent publication (3). The example on the DGLinker website shows the results obtained using the ALS associated genes from DisGeNet as input known disease genes, and the most recent versions of DisGeNet, IntAct and Gene Ontology.

## DISCUSSION

As our understanding of disease grows, it becomes possible to predict missing DG links with increasing accuracy. The DGLinker webserver aims to make this predictive capability widely available by automating data pre-processing, providing a range of data and allowing the results to be analysed directly. Although there have been a number of studies to date predicting DG association, DGLinker is the only current webserver tool to automate this increasingly powerful process while providing the necessary flexibility and making the results available for downstream validation.

In some cases, DGLinker does not make new predictions. The primary reasons are a lack of sufficient training data or limited overlap of the selected datasets. In these cases, we recommend adjusting the selection of databases and input genes accordingly.

At present DGLinker includes a number of datasets and analysis tools. We will continue to develop the platform making additional databases and methods for the analysis of the results available. To this end, we would welcome requests of specific databases and tools by the users. Considering the heterogeneity of the genetic architecture and of the underlying biology of human diseases, we recognise the importance of in-depth testing of the method for specific diseases. Following our work on ALS (3), we will perform studies on single or subgroups of diseases, to explore the performance of DGLinker and provide guidance and custom protocols for such cases via new tutorials on the website or open-access publications.

## Supplementary Material

gkab449_Supplemental_File
