# Supplementary material for: DGLinker: flexible knowledge-graph prediction of disease–gene associations
Source: Nucleic Acids Res. 2021 Jun 14;49(W1):W153–61. doi: 10.1093/nar/gkab449 (PMC8262728; doi:10.1093/nar/gkab449)
Supplement: gkab449_Supplemental_File [file gkab449_supplemental_file.pdf]

Supplementary data of:

DGLinker: flexible knowledge-graph prediction of disease-gene associations

Jiajing Hu[1,2,*], Rosalba Lepore[3,*], Richard Dobson[1,4,5], Ammar Al-Chalabi[2,6], Daniel Bean[1,4,*], and Alfredo Iacoangeli[1,2,7,*,#]
[1] Department of Biostatistics and Health Informatics, Institute of Psychiatry, Psychology & Neuroscience, King's College London, London, UK; [2] Department of Basic and Clinical Neuroscience, Maurice Wohl Clinical Neuroscience Institute, Institute of Psychiatry, Psychology & Neuroscience, King's College London, London, UK; [3]BSC-CNS Barcelona Supercomputing Center, Barcelona, Spain; [4]Health Data Research UK London, University College London, London, UK; [5] Institute of Health Informatics, University College London, London, UK; [6] King's College Hospital, Bessemer Road, Denmark Hill, Brixton, London SE5 9RS, London, UK; [7]National Institute for Health Research Biomedical Research Centre and Dementia Unit at South London and Maudsley NHS Foundation Trust and King's College London, London, UK.

Keywords: knowledge-graph, gene prioritization, machine learning, human disease.

*These authors contributed equally
#Correspondance should be addressed to alfredo.iacoangeli@kcl.ac.uk

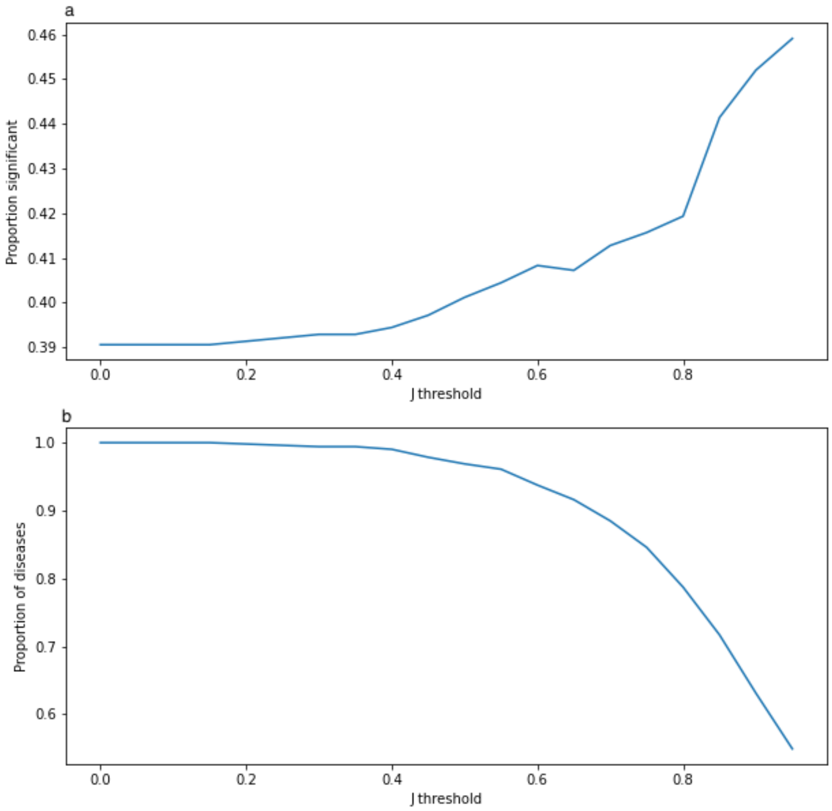

Supplementary figure 1. Effect of J-statistic threshold on model validation and number of models. Analysis performed for all 512 diseases for which the external validation had sufficient statistical power. A) proportion of models with training J >= threshold that are significant. B) proportion of models retained with training J >= threshold.

J threshold	Proportion significant at threshold	Number at threshold	Proportion at threshold
0.00	0.39	512	1.00
0.05	0.39	512	1.00
0.10	0.39	512	1.00
0.15	0.39	512	1.00
0.20	0.39	511	1.00
0.25	0.39	510	1.00
0.30	0.39	509	0.99
0.35	0.39	509	0.99
0.40	0.39	507	0.99
0.45	0.40	501	0.98
0.50	0.40	496	0.97
0.55	0.40	492	0.96
0.60	0.41	480	0.94
0.65	0.41	469	0.92
0.70	0.41	453	0.88
0.75	0.42	433	0.85
0.80	0.42	403	0.79
0.85	0.44	367	0.72
0.90	0.45	323	0.63
0.95	0.46	281	0.55

Supplementary table 1. Effect of J-statistic threshold on validation performance. Analysis performed for all 512 diseases for which the external validation had sufficient statistical power. Models are retained if training J >= threshold for each row.
